# Effects of a *Cc2d1a/Freud-1 Knockdown* in the Hippocampus on Behavior, the Serotonin System, and BDNF

**DOI:** 10.3390/ijms222413319

**Published:** 2021-12-11

**Authors:** Elena M. Kondaurova, Alexandra V. Plyusnina, Tatiana V. Ilchibaeva, Dmitry V. Eremin, Alexander Ya. Rodnyy, Yulia D. Grygoreva, Vladimir S. Naumenko

**Affiliations:** The Federal Research Center Institute of Cytology and Genetics (ICG), Siberian Branch of Russian Academy of Sciences (SB RAS), 630090 Novosibirsk, Russia; ilchibaeva@bionet.nsc.ru (T.V.I.); eremin@bionet.nsc.ru (D.V.E.); arodnyi@bionet.nsc.ru (A.Y.R.); grigorevaulia@bionet.nsc.ru (Y.D.G.); naumenko2002@bionet.nsc.ru (V.S.N.)

**Keywords:** *Cc2d1a/Freud-1* *knockdown*, AAV, 5-HT, 5-HT_1A_ receptor, mouse, CREB, BDNF

## Abstract

The serotonin 5-HT_1A_ receptor is one of the most abundant and widely distributed brain serotonin (5-HT) receptors that play a major role in the modulation of emotions and behavior. The 5-HT_1A_ receptor gene (*Htr1a*) is under the control of transcription factor Freud-1 (also known as *Cc2d1a/Freud-1*). Here, using adeno-associated virus (AAV) constructs in vivo, we investigated effects of a *Cc2d1a/Freud-1 knockdown* in the hippocampus of C57BL/6J mice on behavior, the brain 5-HT system, and brain-derived neurotrophic factor (BDNF). AAV particles carrying the pAAV_H1-2_shRNA-Freud-1_Syn_EGFP plasmid encoding a short-hairpin RNA targeting mouse *Cc2d1a/Freud-1* mRNA had an antidepressant effect in the forced swim test 5 weeks after virus injection. The knockdown impaired spatiotemporal memory as assessed in the Morris water maze. pAAV_H1-2_shRNA-Freud-1_Syn_EGFP decreased *Cc2d1a/Freud-1* mRNA and protein levels. Furthermore, the *Cc2d1a/Freud-1 knockdown* upregulated 5-HT and its metabolite 5-hydroxyindoleacetic acid but not their ratio. The *Cc2d1a/Freud-1 knockdown* failed to increase mRNA and protein levels of *Htr1a* but diminished a 5-HT_1A_ receptor functional response. Meanwhile, the *Cc2d1a/Freud-1 knockdown* reduced *Creb* mRNA expression and CREB phosphorylation and upregulated *cFos* mRNA. The knockdown enhanced the expression of a BDNF precursor (proBDNF protein), which is known to play a crucial part in neuroplasticity. Our data indicate that transcription factor *Cc2d1a/Freud-1* is implicated in the pathogenesis of depressive disorders not only via the 5-HT_1A_ receptor and transcription factor CREB but also through an influence on BDNF.

## 1. Introduction

It is well known that brain serotonin (5-HT) plays an important role in the regulation of many physiological processes and is involved in the control of various types of normal and pathological behaviors, including depression [1,2,3,4]. Numerous studies show a key function of the serotonin 5-HT_1A_ receptor in the regulation of the functional state of the brain 5-HT system [4]. In this regard, this receptor has attracted the attention of many researchers. This receptor in midbrain raphe nuclei acts as a somatodendritic autoreceptor inhibiting neuronal activity and 5-HT secretion into the synaptic cleft. In addition, 5-HT_1A_ receptors are localized postsynaptically. The postsynaptic and presynaptic 5-HT_1A_ receptors affect neuronal activity differently [4,5]. Substantial data indicate the involvement of the 5-HT_1A_ receptor in the pathogenesis of depression [6,7,8,9,10,11], depressive psychosis [12], and suicidal behavior [13,14,15] as well as in the mechanisms of antidepressant-drug action [11,16]. Alterations of the 5-HT_1A_ receptor level are commonly observed in depressed individuals. In particular, the postsynaptic 5-HT_1A_ receptor is downregulated in several cortical regions in depression [17,18,19,20,21] and anxiety [22,23,24], whereas the 5-HT_1A_ autoreceptor is overexpressed in depression [14,25,26]. In this context, elevated 5-HT_1A_ autoreceptor expression tends to reduce the activity of 5-HT neurons, while underexpression of the postsynaptic 5-HT_1A_ receptor may blunt a behavioral response to 5-HT. These data indicate that the 5-HT_1A_ receptor is a major determinant of predisposition to mental disorders. 

It is acknowledged that alterations in the 5-HT_1A_ gene expression can differentially affect the functional activity of the brain 5-HT system via various effects on pre- and postsynaptic 5-HT_1A_ receptors. In view of crucial implication of the 5-HT_1A_ receptor in the autoregulation of brain 5-HT system [4], factors underlying the 5-HT_1A_ receptor gene expression adjustment could be among potential targets for 5-HT-dependent behavior regulation. Nevertheless, mechanisms for 5-HT_1A_ receptor functional state control are still far from being completely elucidated.

In 2000, in the promoter of the gene coding for the 5-HT_1A_ receptor, a binding site (DRE element) for a specific repressor of this gene was detected [27]. Later, transcription factor Freud-1 (encoded by the *Cc2d1a/Freud-1* gene) was identified, which binds to the DRE element in the promoter of the 5-HT_1A_ receptor gene thereby suppressing this gene’s expression in the brain [28]. It is now known that *Cc2d1a/Freud-1* and *Cc2d1b/Freud-2* (a homolog of *Cc2d1a/Freud-1*) mediate dual repression of 5-HT_1A_ receptor expression in most cell types, including many postsynaptic neurons. On the basis of its activities in RN46A cells and its localization in 5-HT neurons in vivo, *Cc2d1a/Freud-1* is believed to serve as a dominant repressor of 5-HT_1A_ autoreceptor expression [6]. Studies on the role of *Cc2d1a/Freud-1* gene overexpression in the 5-HT system suggest that this gene’s product potentially plays a part in the regulation of 5-HT_1A_ receptors in anxiety and major depression [28]. Knockouts of the *Cc2d1a/Freud-1* gene have been shown to be lethal [29,30,31]. In *cF1ko* mice (featuring a conditional knockout of *Cc2d1a/Freud-1* in 5-HT neurons), the loss of *Cc2d1a/Freud-1* in raphe nuclei in adult animals leads to overexpression of 5-HT_1A_ autoreceptors and correlates with higher 5-HT_1A_ receptor functional activity and with lower 5-HT levels. *cF1ko* mice show anxiety- and depression-like behavior that is resistant to chronic treatment with an antidepressant (fluoxetine) [32].

It has been suggested that alterations in transcriptional regulation of the 5-HT_1A_ receptor gene may underlie its dysregulation in mental disorders. Albert and coauthors have proposed that transcriptional downregulation of 5-HT_1A_ autoreceptors is among the reasons for the 3-week delay in the clinical response to antidepressant treatment [16,33,34]. Consistently with this hypothesis, transgenic mice with 30% repression of 5-HT_1A_ autoreceptors display an enhanced and rapid response to selective serotonin reuptake inhibitors (SSRIs) [11], suggesting that transcriptional repression of the 5-HT_1A_ autoreceptor is key to an effective clinical response to an antidepressant [6]. Nevertheless, until now, the 5-HT_1A_ receptor has not been regarded as an independent target of new-generation antidepressants.

On the other hand, it is well known that the majority of prescribed antidepressants (upon chronic use) are capable of increasing brain-derived neurotrophic factor (BDNF) levels, which is apparently a part of their therapeutic action. The mechanisms of this phenomenon remain unknown [35]. The 5-HT_1A_ receptor may be among the drug targets responsible for the antidepressant-induced overexpression of BDNF. These assumptions are consistent with evidence of a positive (therapeutic) effect of 5-HT_1A_ receptor activation by antidepressants (e.g., vilazodone) on the BDNF level in depressive disorders [36]. BDNF is synthesized as a precursor, preproBDNF, in the endoplasmic reticulum; then, it is processed and transported as proBDNF to the Golgi apparatus, where after proteolytic cleavage, it is converted into mature BDNF with simultaneous removal of a pro-peptide [37,38]. ProBDNF can also be secreted by the cell independently. Both proBDNF and BDNF exert functional actions, although BDNF binds to receptor TrkB and causes protein synthesis, axon growth, dendrite maturation, and synaptic-plasticity enhancement [38], whereas proBDNF inhibits neurite growth, reduces neuron size, and initiates apoptosis processes through receptor p75^NTR^ via JNK [39,40].

The cross-talk between the 5-HT system and BDNF seems to be crucial for their functioning and regulation of various physiological processes [41,42]. It can be assumed that the inter-relation between the 5-HT system and BDNF also involves *Cc2d1a/Freud-1* because the latter is a transcriptional regulator not only for the 5-HT_1A_ receptor gene but also for genes of many other factors, such as NF-kappa B (NF-κB) [43] and CREB (cAMP response element–binding protein) [44]. proBDNF activates NF-κB via receptor p75^NTR^, promoting cell survival [45]. At the same time, it is known that NF-κB regulates the transcription of the 5-HT_1A_ receptor gene [46]. Additionally, BDNF initiates ERK-dependent activation of nuclear transcription factor CREB via receptor TrkB [47].

Based on these data, the present study was designed to further clarify the molecular mechanisms of *Cc2d1a/Freud-1* involvement in the regulation of the 5-HT_1A_ receptor as well as the role of *Cc2d1a/Freud-1* in behavior and its possible participation in the cross-talk between the 5-HT system and BDNF in C57BL/6J mice. For this purpose, we investigated the impact of a *Cc2d1a/Freud-1 knockdown* in the hippocampus using adeno-associated virus (AAV) particles carrying a plasmid encoding a small hairpin RNA (shRNA) targeting *Cc2d1a/Freud-1* mRNA.

## 2. Results

### 2.1. The First Set of Experiments (3 Week of Recovery after AAV Administration)

#### 2.1.1. The Open Field Test

**Figure 1:**
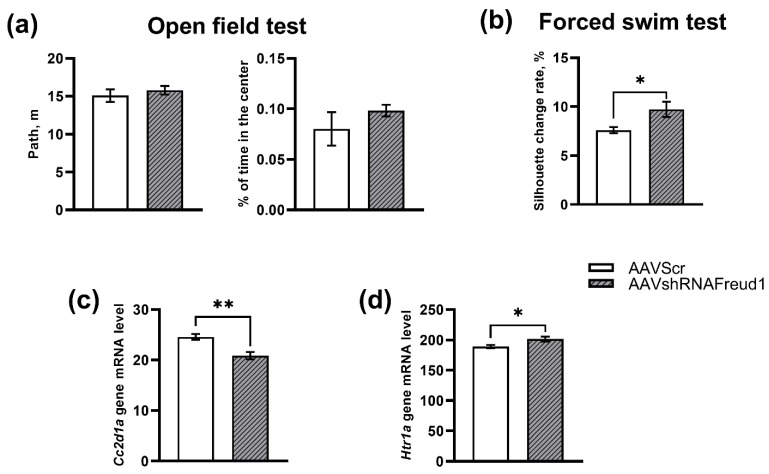
*Cc2d1a/Freud-1* downregulation in the hippocampus of mice did not affect locomotor activity and anxiety-like behavior in the open field test (**a**) while manifesting a significant antidepressant activity in the FST (**b**) 3 weeks after stereotactic injection. *Cc2d1a/Freud-1* (**c**) and *Htr1a* (**d**) mRNA levels in the hippocampus of the experimental mice. Gene expression is presented as the number of cDNA gene copies per 100 cDNA copies of *Polr2a*. * *p* < 0.05; ** *p* < 0.01. N = 7–9.

The *Cc2d1a/Freud-1 knockdown* in the hippocampus failed to affect the locomotor activity of mice (F_1,16_ = 0.49, *p* = 0.49) and the time spent in the center of the open field (F_1,16_ = 1.06, *p* = 0.32, Figure 1a). Nine animals were tested in each group.

#### 2.1.2. The Forced Swim Test

The immobility time in the FST is the main parameter of a depressive-like state of an animal because the vast majority of clinically effective antidepressants reduce immobility time and increase active attempts to get out [48]. In our study, we utilized the intensity of the active search—estimated as the rate of change in a mouse’s silhouette—as a measure of depressive-like behavior. This parameter negatively correlates with immobility time but is an objective indicator [49]. After 3 weeks of recovery, it was found that the *Cc2d1a/Freud-1 knockdown* significantly raised the rate of change in the silhouette (mobility) of the animals (F_1,11_ = 8.56, *p* < 0.01) as compared to the control group, thus indicating an antidepressant effect of the *Cc2d1a/Freud-1 knockdown* in the hippocampus (Figure 1b). In both groups, eight animals were tested.

#### 2.1.3. mRNA Levels

*Cc2d1a/Freud-1* mRNA underexpression (F_1,13_ = 15.32 *p* < 0.002) was registered in the AAVshRNAFreud1 group compared to the AVVScr mice (Figure 1c). The mRNA level of *Htr1a* encoding 5-HT_1A_ was higher in the AAVshRNAFreud1 group than in AVVScr mice (F_1,13_ = 6.56 *p* < 0.05, Figure 1d). Seven animals were analyzed in the AAVshRNAFreud1 group, and eight animals in the AVVScr group.

As for *Polr2a*, which served as a housekeeping gene (for normalization), we did not find any difference between the groups (F_1,14_ = 1.92, *p* = 0.19).

### 2.2. The Second and Third Sets of Experiments (5 Week of Recovery after AAV Administration)

Next, we evaluated the efficiency of the *Cc2d1a/Freud-1 knockdown* and its ability to change behavior in the FST and in the Morris water maze during a longer period of recovery.

#### 2.2.1. The Open Field Test

**Figure 2:**
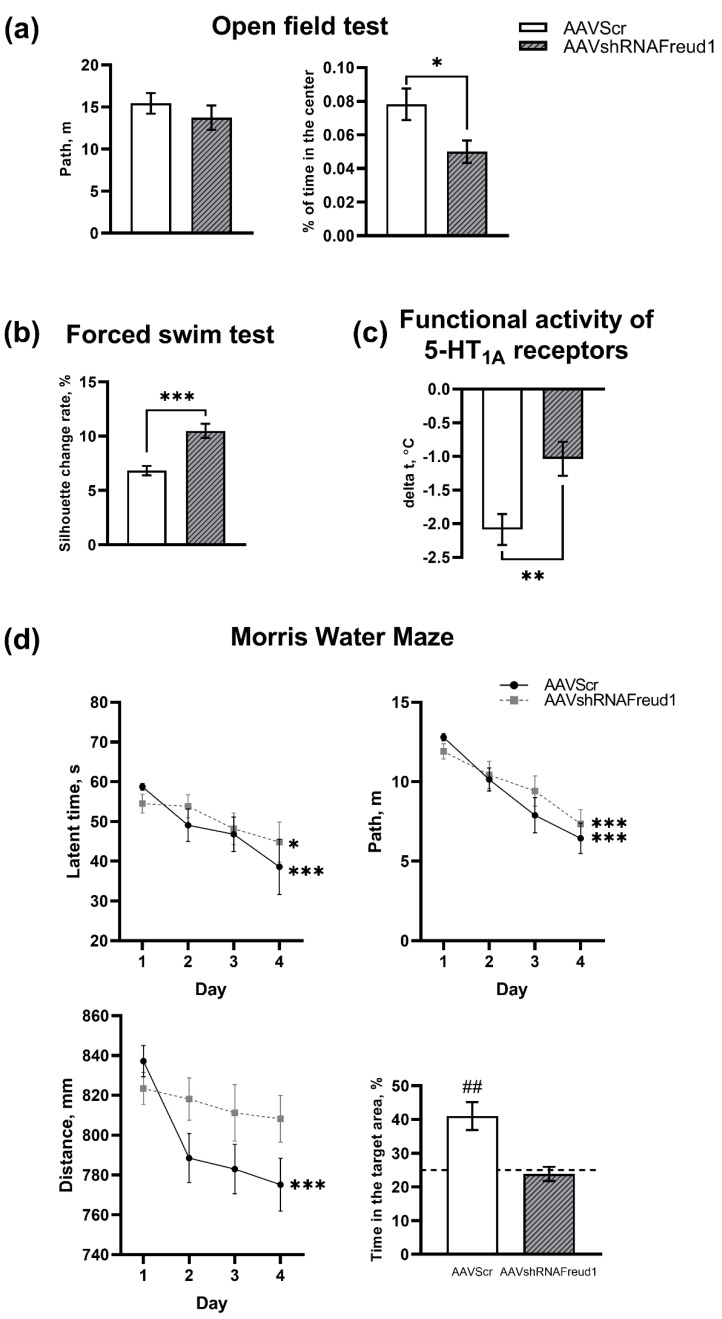
The *Cc2d1a/Freud-1 knockdown* in the hippocampus of mice 5 weeks after the virus injection exerted an anxiogenic effect in the open field test (**a**) and a significant antidepressant action in the FST (**b**); * *p* < 0.05; *** *p* < 0.001. The hypothermic response to acute 8-OH-DPAT administration (1 mg/kg; i.p. injection) was weaker in mice with the *Cc2d1a/Freud-1* suppression (**c**) implying a decrease in 5-HT_1A_ receptor functional activity. ** *p* < 0.01. (**d**) Mice that received AAVshRNAFreud1 failed to find the platform during training and spent less time in the target quarter on the retention day in the Morris water maze test. Dynamics of the cumulative distance alteration between a mouse and the platform, total path, and escape latency during 4 successive days of memory acquisition are presented. * *p* < 0.05; *** *p* < 0.001 vs. the first day of memory acquisition. Time spent in the target sector and in the opposite one in the retest were measured as the means of corresponding two trial values. The horizontal line indicates the chance level. ## *p* < 0.01 vs. the chance level (25%). N = 7–10.

Five weeks after AAV_SynH1-2_shRNA-Freud-1 injection into the hippocampus, we did not notice its influence on the locomotor activity of mice (F_1,18_ = 0.82, *p* = 0.38), thereby confirming results of experiment 1. By contrast, the time spent in the center of the open field was shorter in the AAVshRNAFreud1 group (F_1,17_ = 6.20, *p* < 0.05, Figure 2a). In both groups, ten animals were tested.

#### 2.2.2. The Forced Swim Test

We revealed that the AAVshRNAFreud1 construct led to significant enhancement of the mobility of the animals (F_1,18_ = 21.80, *p* < 0.001) as compared to the control group, meaning an antidepressant action of the Freud-1 knockdown in the hippocampus (Figure 2b). In both groups, 10 animals were tested.

#### 2.2.3. Pharmacological Analysis of the 5-HT_1A_ Receptor Functional Activity

Single i.p. administration of 8-OH-DPAT at a dose of 1 mg/kg (acute stimulation) had a significant hypothermic effect in both studied groups of mice. The 5-HT_1A_ receptor–mediated hypothermic response was weaker in the AAVshRNAFreud1 group (F_1,14_ = 3.47 *p* < 0.05) than in the AVVScr group (Figure 2c). In each group, eight mice were analyzed.

#### 2.2.4. The Morris Water Maze Test

The impact of the knockdown of the 5-HT_1A_ receptor gene silencer on spatiotemporal memory was investigated in the Morris water maze test. The testing showed that mice in the control group achieved successful results on the fourth day of the test, whereas mice in the AAVshRNAFreud1 group continued to fail when trying to find the platform (Figure 2d). 

Repeated-measures ANOVA of the dynamics of memory acquisition uncovered an influence of the test day on the latency of finding the platform (F_3,54_ = 7.84, *p* < 0.01). Moreover, a significant effect of the test day on the distance traveled to the platform (F_3,54_ = 24.82, *p* = 0.002) and the total distance to the platform at each time point (F_3,54_ = 5.67, *p* = 0.002) were documented.

The retention day of the test also showed a memory impairment in mice of the experimental group: mice in the AAVshRNAFreud1 group spent less time in the target quarter (where the platform was previously located) as compared to the control group. In each group, 10 animals were tested.

#### 2.2.5. mRNA, Protein, and Monoamine Levels

**Figure 3:**
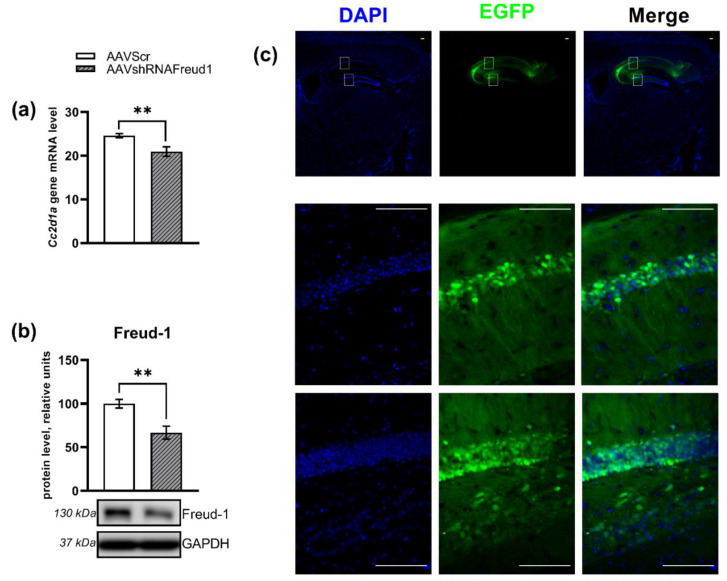
*Cc2d1a/Freud-1* mRNA (**a**) and protein (**b**) levels in the hippocampus of experimental mice 5 weeks after the virus injection. Successful AAV-mediated transfection is indicated by EGFP expression detectable in cells of the CA1 area and the dentate gyrus (**c**). Scale bars are 100 μm. Gene expression is presented as the number of cDNA copies per 100 cDNA copies of *Polr2a*. The CC2D1A/Freud-1 protein level was assessed in chemiluminescence relative units and normalized to GAPDH chemiluminescence relative units. ** *p* < 0.01. N = 8–9.

*Cc2d1a/Freud-1* mRNA underexpression was documented in the AAVshRNAFreud1 group (F_1,15_ = 10.12, *p* < 0.01, Figure 3a) compared to AVVScr animals. An influence of the *Cc2d1a/Freud-1 knockdown* on CC2D1A/Freud-1 protein concentration in the hippocampus (F_1,9_ = 14.74, *p* < 0.005) was also detected in the AAVshRNAFreud1 group compared to AVVScr mice (Figure 3b). Additionally, the expression of plasmids was confirmed by fluorescence microscopy of brain slices (Figure 3c).

**Figure 4:**
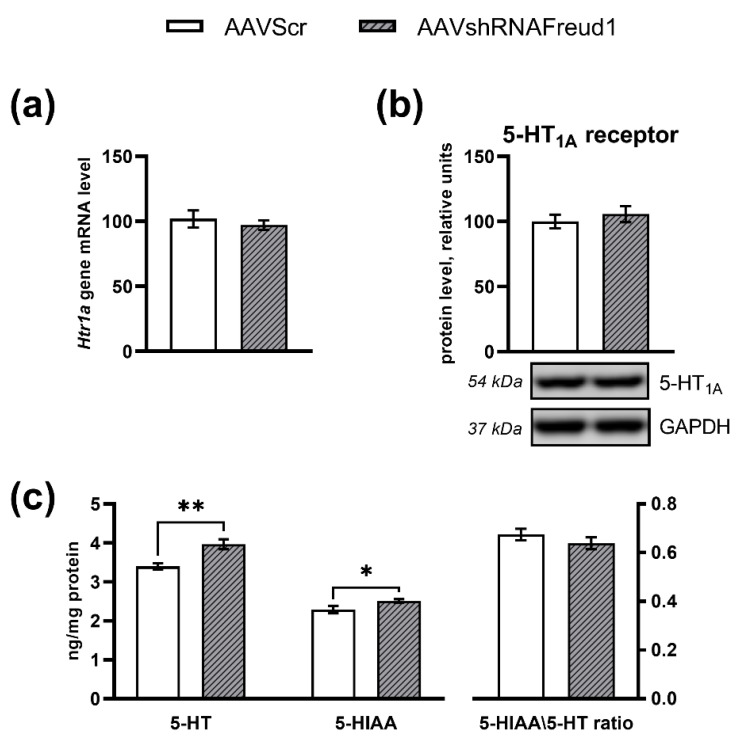
*Htr1a* mRNA level (**a**) and protein (**b**) levels in the hippocampus of experimental and control mice. Gene expression is presented as the number of cDNA copies per 100 cDNA copies of *Polr2a*. Protein levels were assessed in chemiluminescence relative units and normalized to GAPDH chemiluminescence relative units. (**c**) The *Cc2d1a/Freud-1 knockdown* induced an increase in 5-HT and 5-HIAA levels in the hippocampus without changes in the 5-HIAA/5-HT ratio. * *p* < 0.05; ** *p* < 0.01. N = 8–9.

In contrast, the *Cc2d1a/Freud-1 knockdown* failed to affect *Htr1a* mRNA (F_1,15_ = 0.37, *p* > 0.05, Figure 4a) and protein levels (F_1,11_ = 0.12, *p* > 0.05, Figure 4b).

The *Cc2d1a/Freud-1 knockdown* resulted in considerable changes of concentrations of 5-HT and its metabolite 5-hydroxyindoleacetic acid (5-HIAA) in the mouse hippocampus. An increase in the 5-HT level in mice of the AAVshRNAFreud1 group was demonstrated as compared to AAVScr mice (F_1,15_ = 12.99, *p* < 0.05, Figure 4c). The 5-HIAA level was also higher in the AAVshRNAFreud1-treated mice (F_1,15_ = 4.58, *p* < 0.05, Figure 4c). Nevertheless, there was no influence of the *Cc2d1a/Freud-1 knockdown* on the 5-HIAA/5-HT ratio (F_1,15_ = 1.11, *p* > 0.05, Figure 4c).

**Figure 5:**
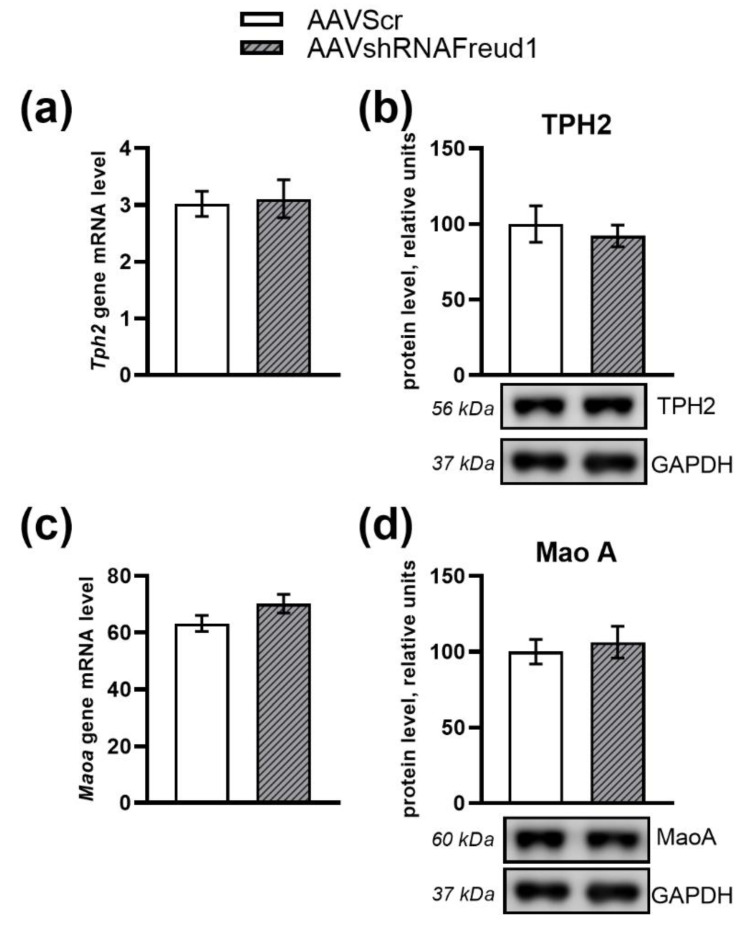
*Tph2* mRNA level (**a**) and protein (**b**) levels. *Maoa* mRNA level (**c**) and protein (**d**) levels in the hippocampus of experimental and control mice. Gene expression is presented as the number of cDNA copies per 100 cDNA copies of *Polr2a*. Protein levels were assessed in chemiluminescence relative units and normalized to GAPDH chemiluminescence relative units. N = 7–9.

The mRNA and protein levels of Tryptophan hydroxylase-2 (*Tph2)* gene did not differ between the two groups (F_1,16_ = 0.05, *p* > 0.05 for *Tph2* mRNA, F_1,12_ = 0.31, *p* > 0.05 for TPH2 protein level, Figure 5a,b). The mRNA and protein levels of Monoamine oxidase A *(Maoa)* gene were not affected either (F_1,17_ = 2.6, *p* > 0.05 for *Maoa* mRNA, F_1,12_ = 0.22, *p* > 0.05 for MAOA protein level, Figure 5c,d).

**Figure 6:**
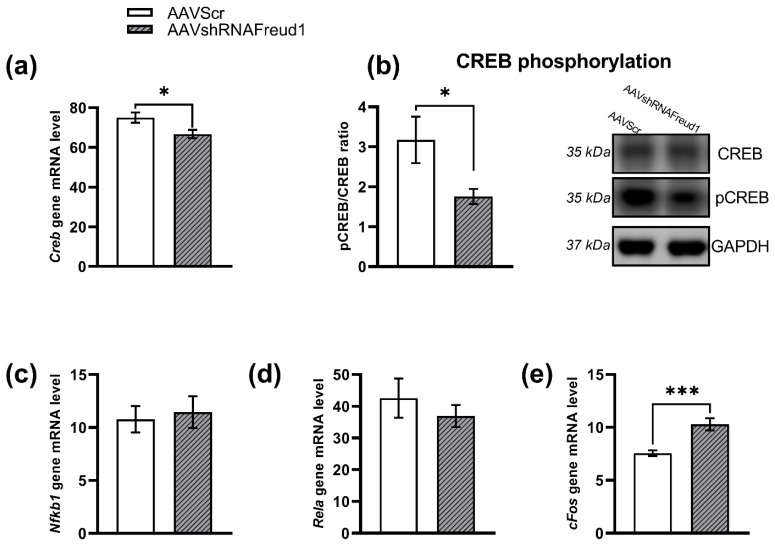
The *Cc2d1a/Freud-1 knockdown* decreased both the *Creb* mRNA level (**a**) and CREB phosphorylation (**b**). At the same time, the mRNA level of *cFos* was higher in the mice with the *Cc2d1a/Freud-1 knockdown* (**e**). mRNA levels of genes *Ntrkb1* (**c**) and *Rela* (**d**) remained unchanged. Gene expression is presented as the number of cDNA copies per 100 cDNA copies of *Polr2a*. Protein levels were assessed in chemiluminescence relative units and normalized to GAPDH chemiluminescence relative units * *p* < 0.05; *** *p* < 0.001. N = 8–9.

The *Creb* mRNA level and CREB phosphorylation were lower in mice of the AAVshRNAFreud1 group (F_1,15_ = 6.08, *p* < 0.05, for *Creb* mRNA and F_1,11_ = 6.11, *p* < 0.05, for CREB phosphorylation, Figure 6a,b), whereas the *cFos* mRNA level was higher in these animals (F_1,15_ = 19.77, *p* < 0.001, Figure 6e). *Rela* (encoding subunit P65 of NFκB factor) and *Nfkb1* (encoding subunit P50 of NFκB factor) mRNA levels were not different between the two groups (F_1,15_ = 0.60, *p* > 0.05, for *Rela* and F_1,15_ = 0.13, *p* > 0.05, for *Nfkb*1, Figure 6c,d).

**Figure 7:**
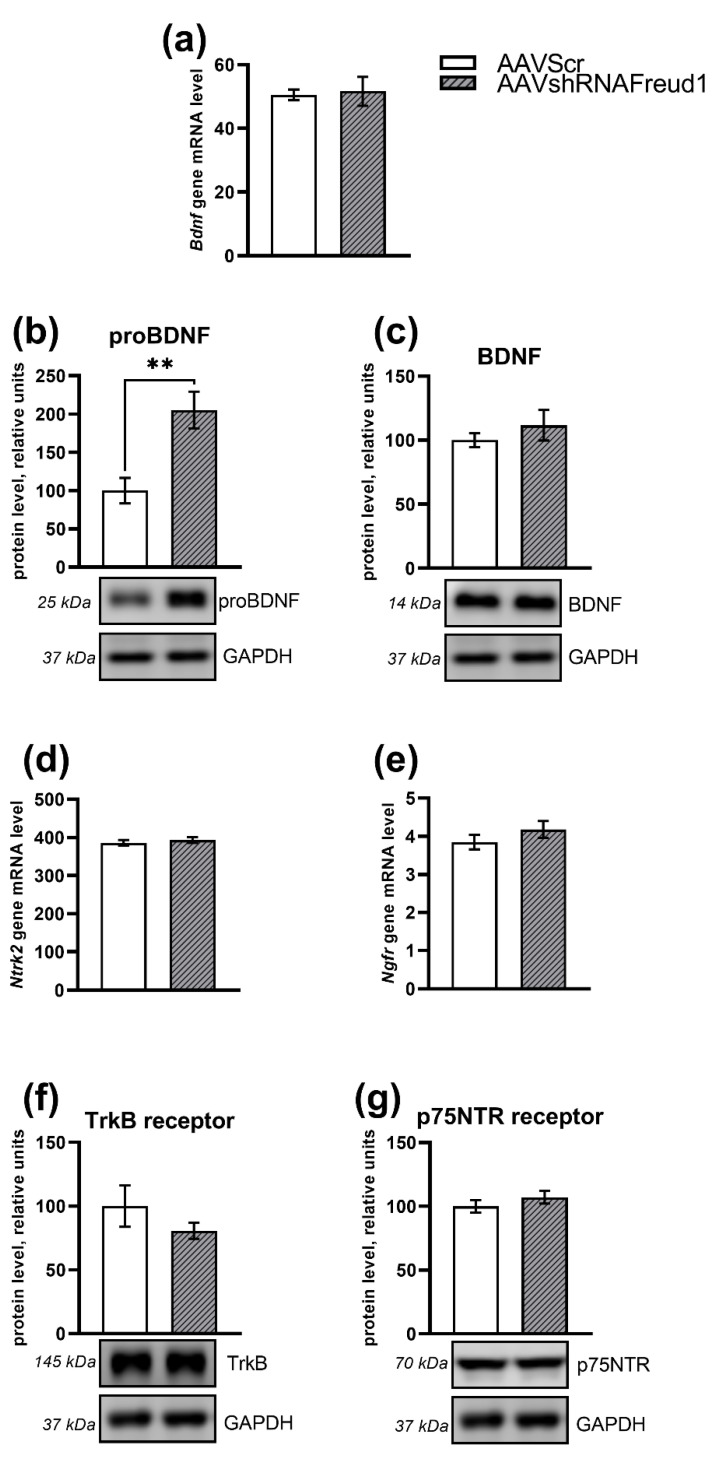
The mRNA level of *Bdnf* (**a**) as well as proBDNF (**b**) and mature BDNF (**c**) protein levels and mRNA levels of *Ntrk2* (**d**) and *Ngfr* (**e**) with protein levels of receptors TrkB (**f**) and p75^NTR^ (**g**) in the hippocampus of the experimental and control mice. Gene expression is presented as the number of cDNA copies per 100 cDNA copies of *Polr2a.* Protein levels were assessed in chemiluminescence relative units and normalized to GAPDH chemiluminescence relative units. ** *p* < 0.01. N = 8–9.

The *Bdnf* mRNA level did not differ between the two groups (F_1,15_ = 0.06, *p* > 0.05, Figure 7a). Nonetheless, it was noticed that the *Cc2d1a/Freud-1 knockdown* upregulated proBDNF (F_1,10_ = 12.9, *p* < 0.005, Figure 7b). There was no effect of the *Cc2d1a/Freud-1 knockdown* on the BDNF protein in the hippocampus (F_1,10_ = 0.81, *p* > 0.05, Figure 7c).

Expression of *Ntrk2* (encoding TrkB) and *Ngfr* (coding for p75^NTR^) did not differ between the two groups (F_1,15_ = 0.42 for *Ntrk2* and F_1,16_ = 1.31 for *Ngfr*, *p* > 0.05, Figure 7d,e). Protein levels of receptors TrkB and p75^NTR^ were not affected either (F_1,10_ = 1.23 for TrkB and F_1,11_ = 1.01 for p75^NTR^, *p* > 0.05, Figure 7f,g). As for *Polr2a*, which served as a housekeeping gene, we did not find any difference between the groups (F_1,16_ = 0.03, *p* = 0.87).

In the qPCR, HPLC assays and Western blot analysis, eight animals were used from the AAVshRNAFreud1 group, and nine animals from the AVVScr group.

## 3. Discussion

Some data indicate that the 5-HT_1A_ receptor is among major drug targets mediating SSRI-induced adult hippocampal neurogenesis: SSRIs do not enhance neurogenesis in 5-HT_1A_ receptor knockout mice [50]. In the present study, we focused on the *Cc2d1a/Freud-1* involvement in the regulation of the 5-HT_1A_ receptor and its function in behavioral plasticity as well as in 5-HT–BDNF cross-talk. For the first time, we showed that *Cc2d1a/Freud-1* downregulation increases mobility in the FST after 3 weeks. Due to classic antidepressants, including those currently prescribed in the clinic, significantly enhancing the mobility of animals in this test [51], the observed CC2D1A/Freud-1-dependent behavioral effect may be considered antidepressive. These behavioral changes were accompanied by a significant reduction in *Cc2d1a/Freud-1* gene expression and, as expected, by *Htr1a* overexpression.

A similar behavioral impact of the *Cc2d1a/Freud-1 knockdown* was observed in the second set of experiments 5 weeks after the AAV administration. In addition, mRNA and protein levels of Cc2d1a/Freud-1 were significantly lower compared to those in the control group. Nonetheless, there were no changes in *Htr1a* mRNA and its protein level after the *Cc2d1a/Freud-1 knockdown*. It can be assumed that during a longer recovery period, a feedback mechanism restores the expression of the 5-HT_1A_ receptor to its baseline. Moreover, other mechanisms and brain systems may be involved in behavior regulation in the FST.

We showed that the partial *Cc2d1a/Freud-1* downregulation in the hippocampus resulted in a spatiotemporal memory disturbance as assessed in the Morris water maze; in the retention test, the mice spent less time in the target quarter. These data are in agreement with the results of Oak et al. and Yang et al. [52,53], who documented a disturbance in spatial learning and memory in mice with a conditional *Cc2d1a/Freud-1* knockout. Several studies imply that the dorsal hippocampus and ventral hippocampus have different functions; the former is involved in cognition, while the latter is more important for the regulation of affective states [54]. For instance, dorsal hippocampus lesions in rats worsen spatial memory in the Morris water maze and radial arm maze [55,56]. Meanwhile, it has been reported that systemic administration of 8-OH-DPAT impairs spatial memory acquisition in the Morris water maze [57,58], and this alteration is abrogated by 5-HT_1A_ receptor antagonists [59,60,61]. Stimulation of the 5-HT_1A_ receptor by 8-OH-DPAT infusion into the CA1 region of the dorsal hippocampus impairs spatial discrimination in rats [60]. Therefore, our results on the *Cc2d1a/Freud-1* suppression–induced impairment of spatiotemporal memory are in good agreement with literature data on the involvement of 5-HT_1A_ receptors in memory and learning mechanisms [62].

We found that in the AAVshRNAFreud1 group, levels of 5-HT and its metabolite 5-HIAA were higher, although the 5-HIAA/5-HT ratio reflecting serotonin catabolism was unchanged. It is obvious that the absence of an influence on the 5-HIAA/5-HT ratio is attributable to enhanced 5-HT catabolism reflected in the higher 5-HIAA level. Overall, our results are consistent with the reports of initial 5-HT upregulation and a return to baseline or even lower levels after prolonged SSRI treatment [63,64,65]. It is known that SSRIs can raise the extracellular level of 5-HT by inhibiting its reuptake into the presynaptic cell, thereby leading to better neurotransmission and improved mood [66]. Similarly, it is possible that the positive impact of the *Cc2d1a/Freud-1 knockdown* in the hippocampus on the behavior in the FST is mediated by 5-HT_1A_ receptor–dependent changes in serotonin reuptake or via altered activity of a catabolic enzyme since no changes were found in the expression of the tryptophan hydroxylase-2 (key enzyme for 5-HT biosynthesis in the brain) and the monoamine oxidase type A (enzyme for 5-HT degradation).

Furthermore, we propose that at 3 weeks after the AAV administration, the knockdown of the *Cc2d1a/Freud-1* gene has an antidepressant effect reflected in higher mobility in the FST via overexpression of the *Htr1a* gene in the hippocampus. In 5 weeks, the antidepressant action persisted due to the higher level of 5-HT and most likely because of the decreased functional response of the 5-HT_1A_ presynaptic autoreceptor. Although the 5-HT_1A_ receptor–mediated hypothermic response is believed to be mediated by the postsynaptic 5-HT_1A_ receptor [67], there is some evidence for the participation of 5-HT_1A_ autoreceptors in the mechanisms underlying 8-OH-DPAT–induced hypothermia [68,69]. Moreover, it has been suggested that the 5-HT_1A_ receptors-mediated hypothermia may be related to those involved in depression and anxiety [70]. It has been demonstrated that rats of the HDS (high 8-OH-DPAT sensitivity) strain exhibit more pronounced immobility in the FST [71] and heightened anxiety in some other tests [72,73]. Chronic antidepressant treatment downregulates 5-HT_1A_ receptor function, judging from 8-OH-DPAT–induced hypothermia [74,75].

Attenuated 8-OH-DPAT-induced hypothermic response observed in mice from *Cc2d1a/Freud-1 knockdown* group was not accompanied by corresponding changes in 5-HT_1A_ receptor expression that is not much of a surprise since 5-HT_1A_ receptor function is depend on a dynamic balance of their production, activation, internalization, and recycling to the plasma membrane in inactivated (desensitized) form [4,76]. Additionally, 5-HT_1A_ receptor is known to be modified posttranslationally [77] and could interact with other 5-HT receptors [78,79,80] that greatly affect receptor function. Moreover, a previous study has suggested that activation of 5-HT_1A_ receptor in the preoptic anterior hypothalamus may be important in the induction of hypothermia [81]. The current data suggest that hippocampal 5-HT_1A_ receptors may be important for thermoregulation as well.

The involvement of the brain 5-HT system and BDNF in the mechanisms behind depressive behavior as well as their mutual regulation are well established [41,42]. BDNF is thought to be intimately involved in the pathogenesis of many disorders of the nervous system, including depression [82,83,84,85,86,87,88]. Here we found that the knockdown of *Cc2d1a/Freud-1* in the hippocampus increased the proBDNF level. The results of our study suggest that BDNF is implicated in the antidepressant-like response (induced by hippocampal CC2D1A/Freud-1 downregulation) through mechanisms involving other transcription factors such as CREB. The latter is one of the key players in the control of expression of immediate early genes. The CRE (Ca^2+^/cAMP-responsive element) site is present in the promoter regions of almost all neuronal immediate early genes including *cFos* and *Bdnf* [89]. *Bdnf* transcription is directly enhanced by CREB [90]. However, here we revealed that the knockdown of *Cc2d1a/Freud-1* in the hippocampus reduces the expression of the *Creb* gene and CREB phosphorylation at the protein level. Our findings are consistent with the data of Zamarbide and coauthors who demonstrated that *Cc2d1a/Freud-1* deficiency in male mice leads to a reduction in CREB signaling [44]. They have also demonstrated that CC2D1A/Freud-1 regulates CREB activation in hippocampal neurons by increasing activity of phosphodiesterase PDE4D, an enzyme involved in cAMP degradation [44]. Earlier, it was shown that spatial memory deficits have been linked to reduced CREB activation in the hippocampus [91]. Together these results explain our data on the *Cc2d1a/Freud-1 knockdown*-induced spatiotemporal learning and memory impairment.

cFos is considered a marker of plastic changes during memory formation because its activation is triggered by higher neuronal activity, and its products play an essential role in plasticity [92]. We noted a higher *cFos* mRNA level after the *Cc2d1a/Freud-1 knockdown* in the hippocampus. It can be assumed that this increase is a response to CREB underexpression or/and could be related to proBDNF protein upregulation. Since *Cc2d1a/Freud-1 knockdown* increased proBDNF level without affecting its mRNA, it can be suggested that *Cc2d1a/Freud-1* may affect (via CREB or other pathways) enzymes involved in the proBDNF processing from preproBDNF. On the other hand, it may be directly related to a decrease of 5-HT_1A_ receptors’ functional activity because their activation is reported to diminish the cFos level [93]. 

It is known that *Cc2d1a/Freud-1* encodes a multifunctional signaling factor, CC2D1A/Freud-1, which regulates multiple pathways taking part in neuronal differentiation by linking transmembrane receptors and their downstream effectors, including protein kinase B (PKB/AKT) activators [94] as well as multiple upstream effectors for transcription factor NF-κB activation [95,96]. In turn, NF-κB activation also depends on 5-HT_1A_ receptor triggering [97]. At the same time, proBDNF activates NF-κB via p75^NTR^, promoting cell survival [45]. Nevertheless, we failed to detect an influence of the *Cc2d1a/Freud-1* suppression in the hippocampus on *RelA* and *Nfkb1* mRNA levels, although it had been shown earlier that both a gain and loss of CC2D1A/Freud-1 function activate NF-κB in developing neurons [98]. Our results mean that the knockdown of *Cc2d1a/Freud-1* in the hippocampus is not sufficient to alter the NF-κB signaling pathway in vivo.

## 4. Materials and Methods

### 4.1. Experimental Design

#### 4.1.1. Animals

All experiments were carried out on adult male mice of the C57BL/6J inbred strain. The mice were housed under standard laboratory conditions on a natural light-dark cycle (12 h light and 12 h dark) with free access to water and feed. Two days before behavioral testing, the mice were weighed (their average weight turned out to be ~25 g) and were isolated in individual cages to prevent group effects [99,100]. The cost of keeping animals was supported by basic-research project No. 0259-2021-0015 and was implemented using the equipment of the Center for Laboratory Animal Genetic Resources at the ICG SB RAS with support from the Ministry of Science and Higher Education of the Russian Federation (project No. RFMEFI62117X0015). All experimental procedures complied with the Guide for the Care and Use of Laboratory Animals, Eighth Edition, Committee for the Update of the Guide for the Care and Use of Laboratory Animals (National Research Council © 2011 National Academy of Sciences, Washington, DC, USA).

#### 4.1.2. Plasmids

The sequences of shRNAs against *Cc2d1a/Freud-1* mRNA were as follows: 

sense 5′-gatccccagatacctctgaggctgtcttcaagagagacagcctcagaggtatctttttttttggaaag-3′; antisense 5′-tcgactttccaaaaaaagatacctctgaggctgtctctctctgaagacagcctcagaggtatctggg-3′.

**Figure 8:**
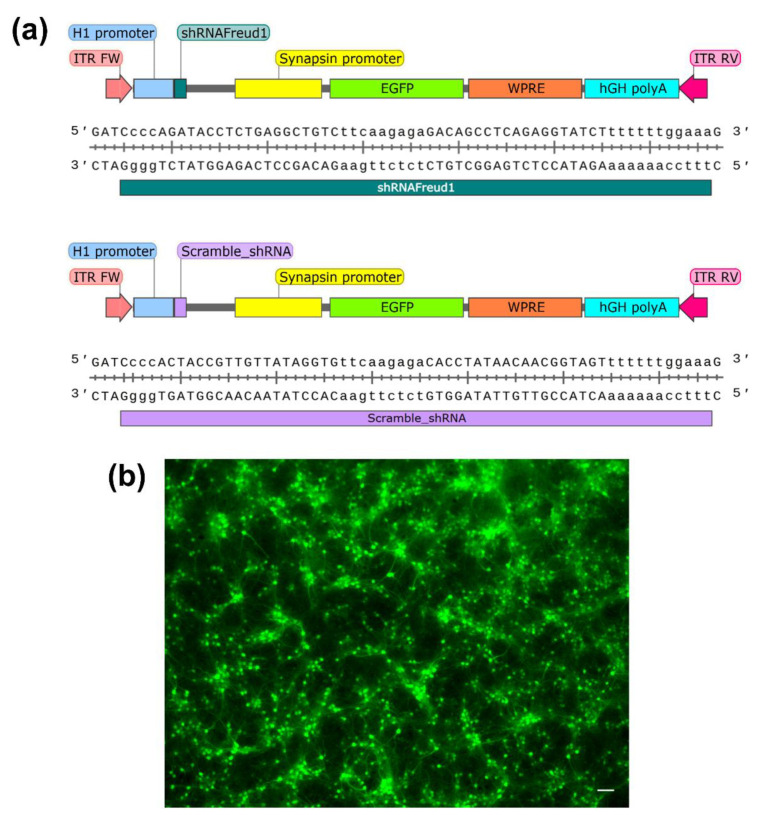
Maps of the AAV vectors (**a**). ITR, inverted terminal repeat; EGFP, enhanced green fluorescent protein gene; Syn, synapsin promoter; WPRE, woodchuck hepatitis virus post-transcriptional regulatory element; hGH polyA, human growth hormone polyadenylation signal. (**b**) AAV-mediated infection as indicated by the EGFP expression is detected in primary hippocampal neurons. Scale bars are 100 μm.

The annealed double-stranded oligo was inserted at BglII and SalI sites in the multiple cloning region of the pAAV-Syn-EGFP-H1-2 vector under the control of the histone H1 promoter [101]. This vector also carries a green fluorescent protein for testing the effectiveness of plasmid expression. All cloning steps were verified by Sanger sequencing. A scrambled shRNA was a gift from Evgeni Ponimaskin (MHH, Hannover, Germany). Both AAV vectors carry the H1 promoter for the expression of either *Cc2d1a/Freud-1* shRNA or the scrambled control shRNA, whereas EGFP expression is driven by the synapsin promoter (Figure 8a).

#### 4.1.3. Cell Culture and Transfection

HEK293FT cells were subcultured in Dulbecco’s modified Eagle’s medium (DMEM) (Sigma–Aldrich, Burlington, MA, USA) supplemented with 10% of fetal bovine serum (FBS, Gibco, Carlsbad, CA, USA), 1% of GlutaMAX (Gibco, Carlsbad, CA, USA), and 1% of Penicillin–Streptomycin (Gibco, Carlsbad, CA, USA). The cells were incubated at 37 °C and 5% CO_2_. The cells were subcultured at 70% confluence, and the medium was refreshed every 2–3 days. HEK293FT cells were transfected with plasmids using polyethylenimine (PEI, 23966-2, Polysciences, Warrington, PA, USA), following the manufacturer’s instructions.

#### 4.1.4. AAV Production

The packaging of pAAV_H1-2_shRNA-Freud-1_Syn_EGFP or of the scrambled-control-shRNA–expressing plasmid into AAV capsids was performed by cotransfection with AAV-DJ and pHelper (Cell Biolabs, Inc., San Diego, CA, USA) plasmids in HEK-293FT cells [101]. Viral particles were harvested after 48 h, according to the protocol described by Grimm et al. [102]. The amount of the obtained viral particles was determined by quantitative PCR (qPCR) with the following primer set: F, 5′-cctggttgctgtctctttatgagg; R, 5′-tgacaggtggtggcaatgc. A series of dilutions of the original plasmid of known concentration served as a standard for determining the number of viral particles. Both AAV vectors used in this study yielded similar genomic titers (10^9^ viral genomes per microliter).

#### 4.1.5. Primary Neuronal Culture

Cultures of hippocampal neurons were prepared from C57BL/6J mice on embryonal day 18 according to an optimized protocol for mouse hippocampal neurons [103]. Briefly, hippocampi were excised and digested by trypsin treatment for 20 min. The obtained cell suspension was centrifuged at 200 g for 3 min. After, dissociated neurons were resuspended in DMEM (Sigma–Aldrich, Burlington, MA, USA) supplemented with 10% of FBS (Gibco, Carlsbad, CA, USA) and plated on 18 mm glass coverslips coated with 0.1 mg/mL poly-d-lysine (Sigma–Aldrich, Burlington, MA, USA). After, 1 h incubation at 37 °C, the Neurobasal-A medium (Gibco, Carlsbad, CA, USA) that contained 20 mM GlutaMAX supplement (Gibco, Carlsbad, CA, USA), B-27 supplement (Gibco, Carlsbad, CA, USA), and 100 U/mL penicillin/streptomycin (Sigma–Aldrich, Burlington, MA, USA) was added. The cultures were maintained at 37 °C in a humidified incubator in an atmosphere containing 5% of CO_2_. Half of the medium was refreshed every third day. Cell transduction with an AAV vector was performed on the ninth day in vitro. The effectiveness of AVV transduction was demonstrated (Figure 8b).

#### 4.1.6. Stereotactic Injection into the Hippocampus

Mice were anesthetized with 400 µL of a 1:1 mixture of 2,2,2-tribromethanol (Sigma–Aldrich, Burlington, MA, USA) and 2-methyl 2-butanol (Sigma–Aldrich, Burlington, MA, USA) diluted 40-fold in saline and placed in a stereotaxic frame (TSE systems, Bad Homburg, Hessen, Germany). Briefly, the scalp was opened, and two holes were drilled in the skull (AP: −1.5 mm, ML: −1.0 mm, DV: 1.5 mm and AP: −1.5 mm, ML: +1.0 mm, DV: 1.5 mm) [104]. The viruses carrying pAAV_H1-2_shRNA-Freud-1_Syn_EGFP (group AAVshRNAFreud1) were slowly bilaterally injected (1 μL per side) into the hippocampus area through a Hamilton syringe at a rate of 0.1 µL/min for 10 min. After the injection, the needle was kept in place for additional 2 min to minimize any drawback of the virus suspension as the needle was removed. As an appropriate control, the AAV expressing the scrambled control shRNA (group AVVScr) was administered in the same way. After the bilateral injections of the virus, the incision was closed with interrupted silk sutures, and the animal was placed in a warm cage and monitored closely.

**Figure 9:**
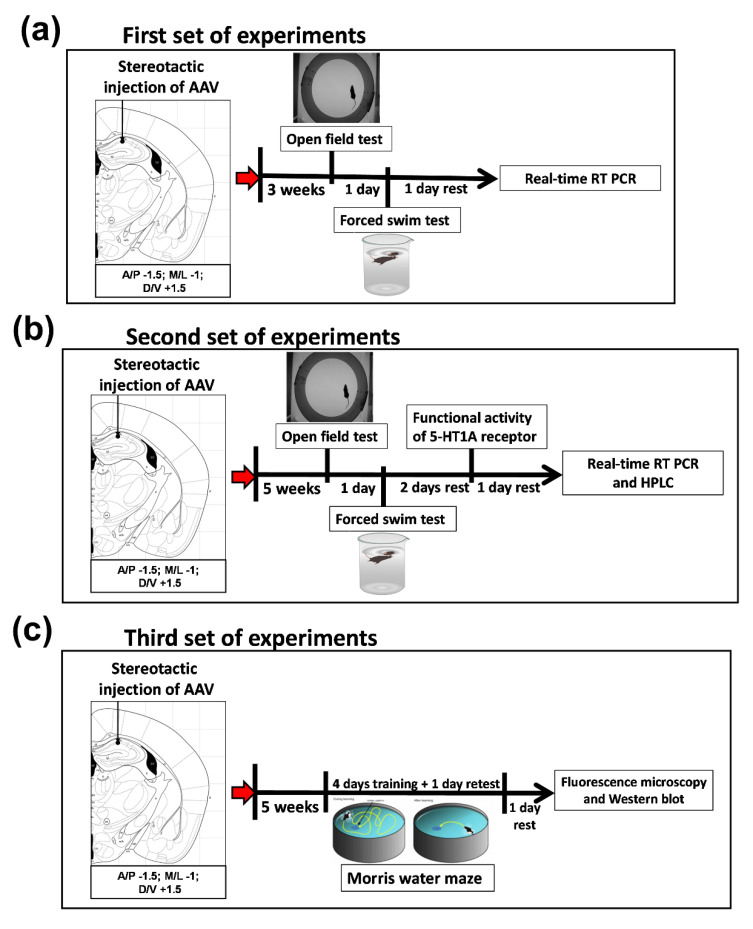
Design of the experiment. (**a**) First set of experiments. (**b**) Second set of experiments. (**c**) Third set of experiments.

In the first set of experiments, after 3 weeks of recovery, we carried out behavioral tests (open field test and forced swim test; FST), fluorescence microscopy and qPCR in order to examine the effect of the plasmid transfection in vivo. Both behavioral tests were conducted with video registration and calculations in the Ethostudio software developed in our laboratory [49]. At least 8 animals from each group were analyzed (Figure 9a).

In the second set of experiments, after 5 weeks of recovery, behavior in the open field test and FST was evaluated (both behavioral tests were conducted with video registration and calculations in the Ethostudio software), a pharmacological analysis of 5-HT_1A_ receptor functional activity was performed, and hippocampus samples were collected for qPCR and high-performance liquid chromatography (HPLC) assays. In each group, at least 10 animals were analyzed (Figure 9b).

In the third set of experiments, after 5 weeks of recovery, the Morris water maze test was conducted with video registration and calculations in the Ethostudio software, and hippocampus samples were collected for Western blot. In each group, at least 10 animals were analyzed (Figure 9c).

#### 4.1.7. Hippocampus Isolation

At 46–48 h after behavioral testing, animals were decapitated, and their hippocampi were excised on ice, frozen in liquid nitrogen, and stored at −80 °C until qPCR, HPLC, and Western blot experiments were carried out. In the two groups, hippocampi were excised on the same day (12:00–14:00 p.m.).

#### 4.1.8. The Open Field Test

This test was carried out in a circular arena (40 cm in diameter) surrounded by a white plastic wall (25 cm high) and illuminated through a mat and semitransparent floor with two halogen lamps of 12 W each placed 40 cm under the floor [105]. Each mouse was placed near the wall and tested for 5 min. The distance traveled and time spent in the center of the arena were measured.

#### 4.1.9. The Forced Swim Test

Each mouse was placed in a clear plastic box (30 × 30 × 30 cm) filled with water of 25 °C. Mouse mobility was measured for 4 min (after 2 min adaptation) by the Ethostudio program. The program measured the rate of change in the silhouette of an animal, which was determined as the number of animal-associated pixels changed between two adjacent frames [106].

#### 4.1.10. Pharmacological Analysis of 5-HT_1A_ Receptor Functional Activity

The functional activity of 5-HT_1A_ receptors was estimated by quantification of the hypothermic response to acute administration of a 5-HT_1A_ agonist: (±)-8-Hydroxy-2-(dipropylamino)tetralin hydrobromide (8-OH-DPAT; at 1 mg/kg intraperitoneally [i.p.]) [71,107]. Core temperature of the mice was measured by means of a KJT thermocouple (Hanna Instruments, Singapore) with a copper constantan rectal probe (Physitemp Instruments, Clifton, NJ, USA) before and 20 min after the drug administration.

#### 4.1.11. The Morris Water Maze Test

The experiment was conducted as previously described [108]. A circular white plastic tank (100 cm diameter, 40 cm high) with a mat and semitransparent floor was employed. We tracked each animal by transmitted (inverted)-lighting techniques developed for the open field test [105]. The water (25 °C) was rendered opaque by the addition of nonfat dry milk. The surface of the tank was virtually subdivided into four quadrants. A glass escape round platform (5 cm diameter and 14.5 cm height) was located 1 cm below the water surface near the center of the target quadrant of the maze. Each mouse underwent three acquisition trials per day for 4 consecutive days. Trial duration was limited to 60 s. During each training trial, an animal was released into the maze from the fixed start position in the center of the tank. Regardless of whether the mouse found the platform, it was placed on the platform and allowed to stay on it for 15 s. Parameters measured during an acquisition trial included latency to reach the hidden platform (escape latency time, s) and the path traveled to the platform (cm). Mean values were calculated from the three trials of the same day [109].

On the fifth day immediately following the above-mentioned 4 days, the retention test was performed. The platform was removed, and the mouse was released into the center of the tank. During two 60 s trials separated by 15 s intervals, the time spent (%) in the target quadrant was measured. Mean values of the two trials were calculated.

#### 4.1.12. Fluorescence Microscopy

At least 6 weeks after AAV injection, mice were transcardially perfused for 2 min with 5 mL of phosphate-buffered saline (PBS) and 25 mL of a 4% paraformaldehyde solution for 10 min under anesthesia. The brain was removed and postfixed with 4% paraformaldehyde for 5 h and immersed in 30% sucrose in PBS for 2 days. Sequential 20 µm slices were prepared on a cryostat (Thermo Fisher Scientific Inc., Waltham, MA, USA). Cell nuclei were stained with a bis-benzimide solution (Hoechst 33258 dye, 5 µg/mL in PBS; Sigma–Aldrich, Burlington, MA, USA). Finally, the sections were mounted in an antiquenching medium (Fluoromount G; Southern Biotechnology Associates, Burlington, NC, USA) followed by examination under a Zeiss AxioImager microscope with 2.5× and 20× air-immersion objectives.

#### 4.1.13. qPCR

The frozen hippocampi were homogenized with TRIzol (Invitrogen, Waltham, MA, USA) according to the manufacturer’s instructions. Total RNA was extracted, and 1 µg of the total RNA was subjected to cDNA synthesis with a random hexanucleotide mixture. The numbers of copies of *Cc2d1a/Freud-1, Htr1a, Maoa, Tph2, Bdnf, Ntrk2, Ngfr, Rela, Nfkb1, Creb*, and *cFos* cDNAs were estimated by SYBR Green qPCR with specific primers (Table 1). We used 50, 100, 200, 400, 800, 1600, 3200, and 6400 copies of genomic DNA as external standards for all the studied genes. The gene expression was presented as the number of cDNA copies per 100 copies of *Polr2a* cDNA [110,111,112].

#### 4.1.14. HPLC

The frozen hippocampi were homogenized in the Potter–Elvehjem homogenizer in 200 µL of 0.6 M HClO4 (Sigma–Aldrich, Burlington, MA, USA) containing 200 ng/mL isoproterenol (Sigma–Aldrich, Burlington, MA, USA) as an internal standard. The homogenate was centrifuged at 12,000× *g* for 15 min at 4 °C for protein precipitation. The supernatants were diluted twofold with ultra-pure water and filtered in a centrifuge tube with a 0.22 µm cellulose acetate filter (Spin-X^®^, Burlington, MA, USA). The pellet was stored at −20 °C until protein quantitation by the Bradford method. Twenty microliters of the filtered supernatant was injected into the loop of the HPLC system containing the following components: an electrochemical detector (750 mV, DECADE IITM Electrochemical Detector; Antec, The Netherlands), a glassy carbon flow cell (VT-03 cell 3 mm GC sb; Antec, The Netherlands), system controller CBM-20A, solvent delivery unit LC-20AD, auto sampler SIL-20A, and degasser DGU-20A5R (Shimadzu Corporation, Japan). Chromatographic separation of substances was carried out by isocratic elution at a flow rate of 0.6 mL/min on a C18 column (5 µm particle size, L × I.D. 75 × 4.6 mm, Luna, Penomenex, Torrance, CA, USA) protected by a C8 security guard (Penomenex, Torrance, CA, USA) cartridge. The mobile phase was a mixture of 90% of 50 mM phosphate buffer (Sigma-Aldrich, Burlington, MA, USA) containing 2% of octanesulfonic acid sodium salt (Chimmed, Moscow, Russia) (pH 3.9) and 10% of methanol (Chimmed, Moscow, Russia). The temperature of the column was stabilized at 40 °C. The amounts (ng) of substances were calculated relative to the internal standard. The concentrations of the substances were expressed in ng/mg of protein (as determined by the Bradford assay).

#### 4.1.15. Western Blot

For assessment of total protein levels, the frozen hippocampi were homogenized in LB buffer (300 mM NaCl, 100 mM Tris-HCl pH 8.4 mM EDTA, 0.2% of Triton X-100, 1 mM Na3VO4, 2 mM PMSF, and a protease inhibitor cocktail), incubated for 60 min on ice, and centrifuged (12,000× *g*, 15 min). Supernatant protein was transferred to a clean tube and kept at −80 °C. Protein concentration was estimated spectrophotometrically using the Pierce BCA Protein Assay Kit (Thermo Fisher Scientific Inc., Waltham, MA, USA) on NanoDrop 2000C (Thermo Fisher Scientific Inc., Waltham, MA, USA) followed by adjustment of the samples to equal concentrations of protein with 2× Laemmli sample buffer. The proteins were denatured by boiling for 10 min at 95 °C. The protein extracts (30 μg per lane for BDNF and proBDNF analyses and 15 μg per lane for the analysis of other proteins: CC2D1A/Freud-1, 5-HT_1A_, MAOA, TPH2, CREB, pCREB, p75^NTR^, and TrkB) were resolved by SDS-PAGE and blotted onto a nitrocellulose membrane (Bio-Rad Laboratories, Inc., Hercules, CA, USA) in a Trans-Blot Turbo Transfer System (Bio-Rad Laboratories, Inc., Hercules, CA, USA). The membranes were blocked in Tris-buffered saline supplemented with 0.05% of Tween 20 (TBST) containing 5% of nonfat dry milk (NFDM-TBST) (for BDNF, proBDNF, CC2D1A/Freud-1, 5-HT_1A_, MAOA, TPH2, p75^NTR^, CREB and TrkB), for pCREB the membrane was blocked in 5% BSA. All membranes were blocked for 1 h, rinsed, and next incubated with primary antibodies (Table 2). After protein detection (as described below), all blots were stripped and then reprobed with an anti-GAPDH antibody as a loading control. For protein detection, the membranes were washed in TBST (5 × 5 min), followed by incubation with a secondary antibody conjugated with horseradish peroxidase. After washing, the blots were treated with the Clarity Western ECL Substrate (Bio-Rad Laboratories, Inc., Hercules, CA, USA) according to the manufacturer’s instructions. Protein bands were detected using a C-DiGit Blot Scanner (LI-COR, Lincoln, NE, USA). Quantification of protein bands was performed by means of ImageStudio (LI-COR, Lincoln, NE, USA). Target protein levels were normalized to GAPDH levels and are presented as a percentage of the control animals’ values.

#### 4.1.16. Statistical Analysis

All values are presented as means ± SEM. Differences were assessed by one-way ANOVA and two-way ANOVA for repeated measures (Morris water maze test) followed by Fisher’s post hoc test. Statistical significance was set to *p* < 0.05. The normality of variances was examined by the Kolmogorov–Smirnov and Shapiro–Wilk tests. For all variances, statistical significance in both tests was assumed at *p* > 0.05, which indicates a normal distribution. The Dixon test was conducted to identify and exclude outliers from the analysis; for relative values, outlier removal from a dataset was performed for the absolute values. The time spent in the quadrants in the retention test was compared with the chance level (25%) by two-tailed Student’s *t* test for a single sample.

## 5. Conclusions

This study shows for the first time that a knockdown of *Cc2d1a/Freud-1* in the hippocampus impairs spatiotemporalmemory and exerts an antidepressant action accompanied by significant changes in both the brain 5-HT system and BDNF. Meanwhile, the *Cc2d1a/Freud-1 knockdown* reduces 5-HT_1A_ receptor functional activity and raises 5-HT and 5-HIAA levels in the hippocampus of C57BL6/J mice. Our data suggest that BDNF and transcription factor CREB are also involved in the mechanism underlying the CC2D1A/Freud-1-dependent antidepressant effect. Moreover, CC2D1A/Freud-1 can affect BDNF function thereby possibly playing a substantial part in the pathogenesis of various mental disorders, including depression.

## Figures and Tables

**Table 1:** The primer sequences, annealing temperatures, and PCR product length.

Gene	Sequence	Annealing Temperature, °C	Product Length, bp
*Htr1a*	F 5′-ctgtgacctgtttatcgccctg-3′ R 5′-gtagtctatagggtcggtgattgc-3′	62	200
*Cc2d1a*	F 5′-gcaaagccgggcaacatcatc-3′ R 5′-tagcagaggtgggtgtagtgg-3′	60	181
*Bdnf*	F 5′-tagcaaaaagagaattggctg-3′ R 5′-tttcaggtcatggatatgtcc-3′	59	255
*Ntrk2*	F 5′-cattcactgtgagaggcaacc-3′ F 5′-atcagggtgtagtctccgttatt-3′	63	175
*Ngfr*	F 5′-acaacacccagcacccagga-3′ R 5′-cacaaccacagcagccaaga-3′	62	171
*Rela*	F 5′-gcacctgttccaaagagcac-3′ R 5′-gagttgtccacagatgccagg-3′	64	93
*Nfkb1*	F 5′-cgtctgtctgctctctctcgac-3′ R 5′-ctcgcctcggtacttctctctc-3′	64	152
*Creb*	F 5′-gctggctaacaatggtacggat-3′ R 5′-tggttgctgggcactagaat-3′	64	140
*cFos*	F 5′-aaagagaaggaaaaactggag-3′ R 5′-cggaaacaagaagtcatcaa-3′	58	264
*Tph2*	F 5′-cattcctcgcacaattccagtcg-3′ R 5′-cttgacatattcaactagacgctc-3′	61	239
*Maoa*	F 5′-atgaggatgttaaatgggtagatgttggt-3′ R 5′-cttgacatattcaactagacgctc-	61	138
*Polr2a*	F 5′-tgtgacaactccatacaatgc-3′ R 5′-ctctcttagtgaatttgcgtact-3′	60	194

**Table 2:** The list of antibodies used and immunodetection conditions.

Antibodies, Manufacturer, Cat. No.	Dilution	Incubation Time, Conditions
Rabbit polyclonal antibody to 5-HT_1A_ protein, Abcam, Cambridge, UK, ab85615	1:1000 in TBST supplemented with 5% milk powder	Night at 4 °C, as a primary antibody
Rabbit monoclonal antibody to CC2D1A/Freud-1 protein, Abcam, Cambridge, UK, ab191472	1:2000 in TBST supplemented with 5% milk powder	Night at 4 °C, as a primary antibody
Rabbit antibody to BDNF protein, Abcam, Cambridge, UK, ab46176	1:1000 in TBST supplemented with 5% milk powder	Night at 4 °C, as a primary antibody
Mouse antibody to pro-BDNF protein, Santa Cruz Biotechnology, Dallas, TX, USA, sc65513	1:250 in TBST supplemented with 5% milk powder	Night at 4 °C, as a primary antibody
Rabbit antibody to p75^NTR^, Abcam, Cambridge, UK, ab38335	1:500 in TBST supplemented with 5% milk powder	2 h at RT, as a primary antibody
Rabbit antibody to TrkB, Abcam, Cambridge, UK, ab18987	1:400 in TBST supplemented with 3% BSA	Night at 4 °C, as a primary antibody
Rabbit monoclonal [E113] antibody to CREB protein (phospho S133), Abcam, Cambridge, UK, ab32096	1:1000 in TBST supplemented with 5% BSA	Night at 4 °C, as a primary antibody
Rabbit polyclonal antibody to CREB protein, Abcam, Cambridge, UK, ab31387	1:1000 in TBST supplemented with 5% BSA	Night at 4 °C, as a primary antibody
Rabbit polyclonal antibody to GAPDH protein, Abcam, Cambridge, UK, ab9485	1:2500 in TBST supplemented with 5% BSA	Night at 4 °C, as a primary antibody
Rabbit polyclonal antibody to MAOA Abcam, Cambridge, UK, ab126751	1:500 in TBST supplemented with 5% milk powder	Night at 4 °C, as a primary antibody
Rabbit polyclonal antibody to TPH2 Abcam, Cambridge, UK, ab184505	1:1000 in TBST supplemented with 5% milk powder	Night at 4 °C, as a primary antibody
Goat anti-rabbit IgG antibody conjugated to horseradish peroxidase, Invitrogen, Waltham, MA, USA, G-21234	1:10000 in TBST supplemented with 5% milk powder	1 h at RT, as a secondary antibody
Goat anti-mouse IgG antibody conjugated to horseradish peroxidase, Abcam, Cambridge, UK, ab6728	1:2000 in TBST supplemented with 5% milk powder	1 h at RT, as a secondary antibody

## Data Availability

The datasets generated during the current study are available from the corresponding author on reasonable request.
